# Elder’s life course theory and person-centered research: A lens for conducting ethical nursing research and mental health nursing practice with older adults aging with the diagnosis of schizophrenia

**DOI:** 10.1111/jpm.12819

**Published:** 2022-01-25

**Authors:** Verónica García Walker

**Affiliations:** The University of Texas at Austin, Austin, Texas, USA

**Keywords:** clinical research, ethics, nursing theory, older adult, schizophrenia

## Abstract

**Introduction:**

Older adults diagnosed with schizophrenia are a vulnerable population owing to the manifestations of their illness, which can include decreased reality orientation, paranoia, hallucinations and delusions. This paper presents ethical principles of vulnerability, veracity, non-maleficence and autonomy for person-centered care in mental health nursing research and practice, focused with the lens of Elder’s life course theory (LCT).

**Aim:**

To present Elder’s LCT as an ethical lens for person-centered care as nurses engage with older adults aging with the diagnosis of schizophrenia in clinical practice and/or research.

**Method:**

Four ethical principles fundamental to nursing research and mental health practice are presented, with Elder’s LCT as a theoretical lens for person-centered care.

**Results:**

A model for ethical research and mental health practice with older adults diagnosed with schizophrenia.

**Discussion:**

Nursing research and mental health nursing practice with an ethical LCT lens for person-centered can help nurses envision, explore and generate interventions to address the special needs of older adults aging with schizophrenia.

**Implications for Practice:**

The use of a LCT lens for person-centered care can encourage nurses in research and mental health practice to seek information collaboratively with older adults diagnosed with schizophrenia in a thoughtful, ethical manner, to inform the improvement of their health outcomes and health policy.

## 1 | INTRODUCTION

Schizophrenia is a major cause of mental-health disability, affecting approximately 21 million people worldwide (National Institute of Mental Health, 2018). Older adults aging with the diagnosis of schizophrenia present a vulnerable population, with a mean life expectancy 20 years shorter than the general population’s. Their vulnerability is due in part to impairments associated with their diagnosis, which can include cognitive, behavioral, linguistic and perceptual issues (American Psychiatric Association, 2013; Angers et al., 2021; Medvedev et al., 2021; National Institute of Mental Health, 2018; Walker, 2021). Thus older adults diagnosed with schizophrenia suffer from multiple physical and psychological comorbidities—high rates of diabetes, cardiovascular disease, substance use and depression (American Psychiatric Association, 2013; Brooks et al., 2019). Their mortality and morbidity have also been specifically related to smoking, lack of physical activity, and unhealthy diets, as well as negative side effects of antipsychotics (Brooks et al., 2019; Crespo-Facorro et al., 2021; Gur et al., 2018; Khan & Rajji, 2019; Nath et al., 2021; Siddi et al., 2019; Toender et al., 2020). In addition, mortality among older adults diagnosed with schizophrenia reflects higher rates of suicide and accidents than in older adults without a schizophrenia diagnosis (Khan & Rajji, 2019).

The vulnerability and lower life expectancies of older adults aging with schizophrenia have been examined in relation to trauma and stress (Cancel et al., 2019; Ruby et al., 2017; Schenkel et al., 2005). Traumatic experiences in these older adults reflect the double stigmatism of aging with a mental illness (Al Jurdi et al., 2014; Gauillard, 2016; Ogden, 2014a, 2014b; Zimmerman et al., 2016), and stressors include their limited or impaired social associations with friends and family along the life course (Burbach, 2018; Fakorede et al., 2020; Robustelli et al., 2017), as well as their physical and psychological comorbidities. Such trauma and stress can occur, for example, when one’s agency or autonomy is removed by placement in court-ordered inpatient hospital environments. The deinstitutionalization movement of the 1960s released many older adults diagnosed with schizophrenia from asylums to the care of community settings with limited resources, with the result that they have actually been *trans-institutionalized* or moved to homeless settings, nursing homes and prisons (Allison-Kolb, 2020; Kim, 2016; Norris, 2017). Such a model of care presents grievous problems, and caregivers report that they are untrained or unequipped to treat this complex population adequately (Gauillard, 2016; Kaldy, 2018; Sorrell, 2017; Walker, 2019; Walker & Walker, 2021).

Given this background, older adults diagnosed with schizophrenia may be especially vulnerable in research and mental health nursing practice settings (American Psychiatric Association, 2013; National Alliance on Mental Illness, 2019; Walker, 2019). Some might argue that it is unethical even to conduct research with such a vulnerable population. Others, however, would argue that not to conduct research with older adults aging with schizophrenia precludes them from the benefits of scientific findings that can inform clinical practice and improve their lives. The failure to conduct such research might be not only non-inclusive but also unjust (Matheson et al., 2012).

Both in conducting research and in mental health nursing practice with older adults aging with schizophrenia, one must therefore strive for a healthy ethical balance. It is important to be protective and thoughtful in addressing this population as we attempt to discover ways to improve their health (Doody & Noonan, 2016; Schrems, 2014). Non-maleficence, the obligation to do no harm, is fundamental to nursing research and mental health nursing practice with older adults aging with schizophrenia, because involvement with this population raises ethical questions, many of which are associated with their vulnerabilities. In this paper, I first present four ethical principles to guide nurses in mental health nursing practice and in nursing research as they engage with older adults aging with schizophrenia. Next, I present details of a lens for person-centered care based on life course theory (LCT), which nurses in mental health nursing practice and nursing research can use to help maintain balance and conduct ethical self-examinations when they engage with older adults aging with schizophrenia and with other vulnerable populations. This lens is based on themes from both Elder’s LCT (Elder, 1996; Elder & Giele, 2009) and a person-centered model adapted from the Alzheimer’s Society (2021) and from Fazio et al. (2018). It is well-established that human subjects must be protected, but in the case of older adults aging with schizophrenia, LCT combined with an adapted Alzheimer’s model offers additional insight to inform research and practice for this unique population.

### 1.1 | Vulnerability, veracity, non-maleficence and autonomy in working with older adults diagnosed with schizophrenia

This paper presents four fundamental principles for working with older adults diagnosed with schizophrenia, drawn especially from the work of Beauchamp and Childress, 2019,^2013^. Beauchamp and Childress (2013) originally defined a framework for bioethical decision making in medicine and health care, consisting of the following main principles: *autonomy*, *non-maleficence*, *beneficence* and *justice*. Additional ethical concepts derived from this framework included *veracity* and *vulnerability* (Beauchamp and Childress, 2019,^2013^; Bracken-Roche et al., 2017 Cunha & Garrafa, 2016). Beauchamp and Childress (2019) argued that these ethical principles should be used in “bioethical discourse and practice” in order to resolve “moral problems” that arise in medicine and healthcare (pp. 9–10). In nursing research and mental health practice, the ethical principles of *vulnerability*, *veracity*, *non-maleficence*, and *autonomy* are commonly encountered and addressed (Baughman et al., 2014; Mergen & Akpinar, 2021; Miller et al., 2008; Morris et al., 2021; Starke et al., 2021).

The *vulnerable* include those hospitalized in institutions, who include many older adults diagnosed with schizophrenia. According to the Belmont Report (National Commission for the Protection of Human Subjects of Biomedical and Behavioral Research, 1979), individuals in institutional settings may be more vulnerable or easy to manipulate in research settings, owing to their illnesses and restricted environments, and they should not be selected in research projects simply for the convenience of research. However, Beauchamp and Childress (2013) argued that paternalistically viewing populations as *vulnerable* may preclude them from the benefits of research, and that this might represent a form of discrimination that could lead to further marginalization and stigmatization.

*Veracity*, or, in the present context, “telling the truth” with participants known for their vulnerability, provides an umbrella of protection for the research participant. That is, it might be tempting for a nurse researcher to speed up the informed consent procedure of a study with older adults diagnosed with schizophrenia in order to expedite a research project, but the ethical fact remains that these individuals are entitled to truth or veracity regarding their study participation just as much as are others in society without such a diagnosis. The time required to accomplish the goal of veracity is irrelevant (Doody & Noonan, 2016).

Often the ethical principles of *non-maleficence* and autonomy may appear to be at odds with each other, but together they can provide strong checks and balances for those working with vulnerable populations. For example, nursing researchers may encounter many roadblocks to conducting research with older adults diagnosed with schizophrenia due to the protective nature of caretakers committed to preventing harm, or *non-maleficence* (White, 2020). Reciprocally, however, protectively disallowing research with this population may preclude helpful findings for their future health care and ignore their rights to *autonomy* if it is their wish to participate (Steenbergen, 2021). Thus, it is important for nursing researchers to consider both their own non-maleficence and the patient’s autonomy when engaging in research with older adults diagnosed with schizophrenia. The evident vulnerability of this population should be of paramount importance, requiring vigilance in working with older adults diagnosed with schizophrenia in mental health settings (Harvey & Rosenthal, 2018). However, the autonomy of this population and their right to receive the benefits of research should also be recognized, thus posing a delicate balance, given their vulnerability, between non-maleficence and autonomy.

**FIGURE 1:**
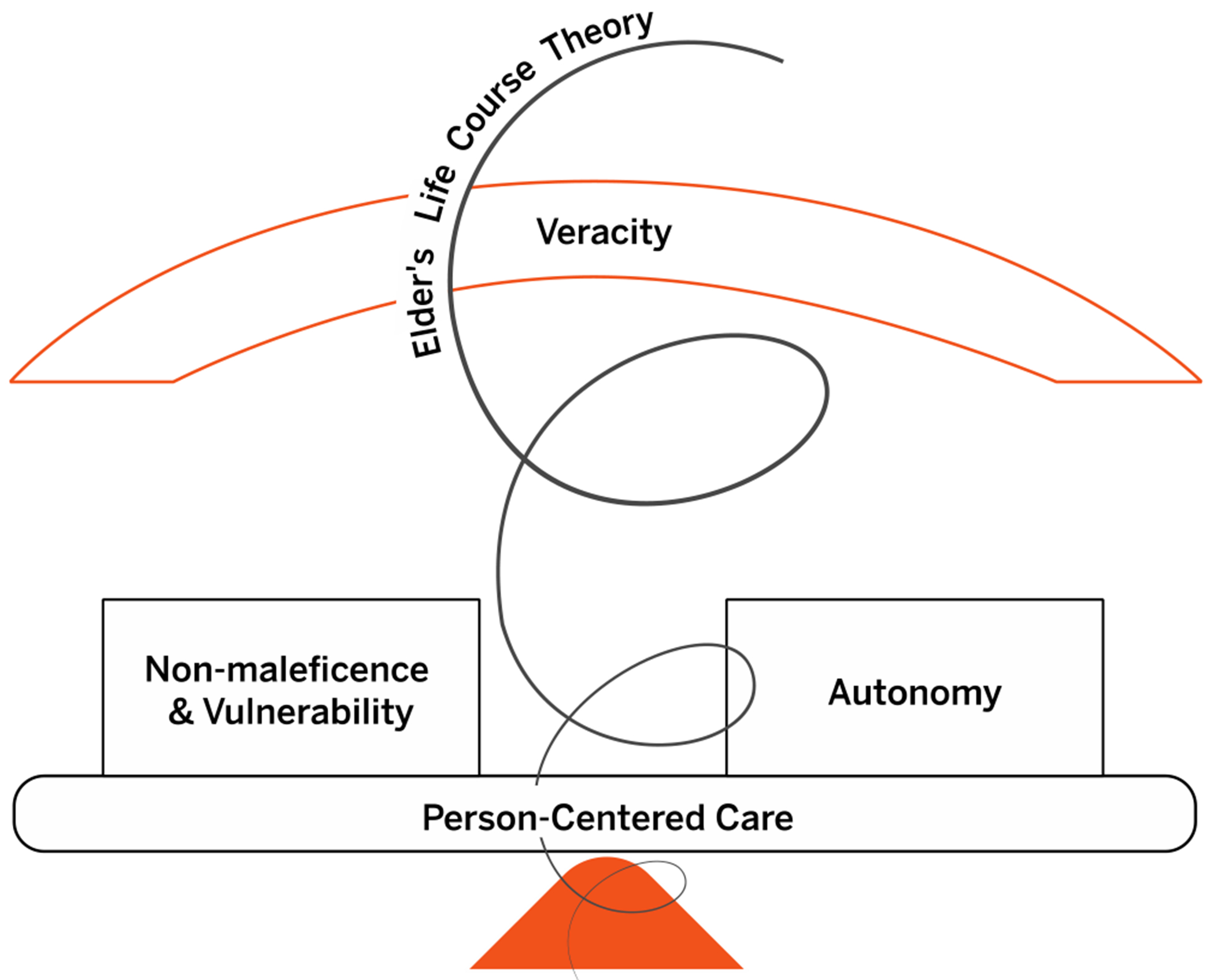
Ethical principle of veracity as an overarching protective umbrella, with the foundation of person-centered care as an underpinning balance for the ethical principles of non-maleficence & vulnerability and autonomy, when conducting nursing research or working in mental health nursing with older adults diagnosed with schizophrenia. Elder’s LCT provides a framework to organize the model holistically

The foundation of *person-centered care* can remind nursing researchers and mental health nurses that the independence, life experiences, culture, reality and autonomy of older adults diagnosed with schizophrenia must be respected and considered within an ethical framework. Vulnerability; veracity, non-maleficence and autonomy, along with a person-centered approach focused with the lens of *Elder’s life course framework* for addressing individuals holistically along the life course, offer the long-term potential to increase holistic, positive patient outcomes for older adults diagnosed with schizophrenia in mental healthcare settings (see Figure 1).

### 1.2 | Vulnerability

Older adults with schizophrenia are a vulnerable population for many reasons. Before deinstitutionalization, society recognized their vulnerability by providing care in asylums (Norris, 2017; Ziff et al., 2008), which included state hospitals. Older adults aging with schizophrenia may have experienced abuse; they may have been treated with stigma and shame. Often they have cognitive deficits that contribute to their risk of being abused (Harvey & Rosenthal, 2018). They may also have limited support systems and lack family members with whom they might associate, thus rendering them alone or nearly alone in facing decisions along the life course, which for our purposes here can include whether or not to become engaged in research or mental health clinical treatments (Burbach, 2018; Robustelli et al., 2017). These potential factors call for thoughtful consideration by the nurse who intends to conduct research or engage in mental health nursing practice with older adults aging with schizophrenia.

### 1.3 | Veracity

In research administration, veracity—telling the truth—can be thought of as a response to darker, less truthful moments in the past when research participants were not told the truth (Doody & Noonan, 2016). In mental health nursing practice and nursing research with older adults aging with schizophrenia, telling the truth means explaining clinical procedures or research projects in a clear, understandable manner whenever these older adults consider participation. Risks must also be described clearly and understandably, so that these participants can provide truly informed consent (Zolnierek, 2007; Zoloth, 2021). LCT reminds us that older adults aging with schizophrenia may not have always met with honest relationships over their life course. Their lives may have included trauma and little social support, resulting in guarded, infrequent trust in individuals whom they encounter in their daily interactions, potentially including mental health nurses and nurse researchers (Burbach, 2018; Robison et al., 2018; Robustelli et al., 2017; Walker, 2019). Veracity is ethically necessary in engagement with older adults aging with schizophrenia in research and in mental health nursing practice.

### 1.4 | Non-maleficence

Non-maleficence, or the obligation to do no harm, is one of the “major moral considerations” and an “essential starting point” for biomedical ethics (Beauchamp and Childress, 2019). This ethical principle is essential to mental health nursing practice and research with vulnerable populations such as older adults aging with schizophrenia. In the past, when research was conducted to investigate problems that were thought important, this was often done in harmful ways (Doody & Noonan, 2016; National Commission for the Protection of Human Subjects of Biomedical and Behavioral Research, 1979; White, 2020). Often, clinical settings also used harmful procedures such as psychosurgeries and insulin comas in attempts to “cure mental illness” (Parsons, 2018, p. 23). Even if many of these procedures may have been well intentioned, they are no longer acceptable; they harm those who are diagnosed with mental illness. In mental health nursing practice and nursing research with older adults aging with schizophrenia, which involves sensitive communication, research questions and interactions with participants or patients can elicit emotional responses with a potential for harm (Doody & Noonan, 2016; van Wijk & Harrison, 2013). With non-maleficence in mind, nurses and nurse researchers can consider potential ill effects associated with communication as they undertake an ethical examination of their engagement with the older adult aging with schizophrenia.

### 1.5 | Autonomy

The necessity of disproportionately juxtaposing the safety of an older adult aging with schizophrenia and the ethical principle of autonomy is frequently encountered by those who engage in mental health nursing practice and research (Steenbergen, 2021; Walker & Walker, 2021). To keep an older adult aging with mental illness safe during times of decompensation and psychosis may require actions that oppose the person’s autonomy or will, because safety in these instances can include court-ordered medications, restraints and seclusions, as well as involuntary hospitalizations (Bauer & Weust, 2017; National Alliance on Mental Illness, 2019; Starr, 2021). The consideration of life course experiences that may have limited the autonomy of older adult patients aging with schizophrenia can help mental health nurses and nurse researchers understand these patients’ sometimes reticent reactions to requests (Walker, 2019). Such requests include their informed consent and participation in clinical procedures as well as research projects.

## 2 | LCT FOR A PERSON-CENTERED ETHICAL NURSING RESEARCH

The sociologist Glen Elder, Jr. was a pioneer in the development of LCT. Elder’s early works examine the influence of historical events such as the great depression on life course trajectories of families and individuals (Elder, 1996; Elder & Giele, 2009; Hutchison, 2019). For nurses and those in other disciplines who seek a more holistic person-centered approach, Elder’s LCT offers an extensive view of ways in which historical experiences and social intersections influence current issues in patients’ health (Walker, 2019). LCT can also inform primary methods of prevention and thus prevent restriction to a simple focus on the resolution of current health problems (Bates et al., 2018; Black et al., 2009). Individualizing treatment in light of a person’s life experiences and intersections is a strong rationale for the use of LCT in developing a model of person-centered care (Bates et al., 2018).

Elder’s LCT also offers a useful model to explain influences along an individual’s life course that determine how the individual perceives and interacts with people in the present; those influences can include mental health nurses and nursing researchers. LCT comprises four basic concepts: *human lives in historical time and place*; *human agency and social constraints*; *linked lives*; and *the timing of lives*. These concepts provide a logical framework for viewing ethical principles in relation to older adults diagnosed with schizophrenia. With LCT, one can organize and pinpoint key times in the life course of individuals when ethical issues may have been salient. For older adults diagnosed with schizophrenia, such key moments may have been *historical in both time and place*; examples include deinstitutionalization and the discovery that antipsychotics medications could decrease symptoms of psychosis (Adams et al., 2014; Samara et al., 2014; Walker, 2019). Other ethical issues are related to human intersections, or *linked lives*, for older adults diagnosed with schizophrenia along the life course. *Human agency and social constraints* along the life course likewise fit well with the study of ethical principles (Elder, 1996; Elder & Giele, 2009; Walker, 2019), and the *timing of lives* in Elder’s life course model emphasizes that along their life course, individuals with schizophrenia often share common timings for social events such as graduating from school, marrying and having children that differ from such timings for others in society (American Psychiatric Association, 2013). These timings, which are due to manifestations of their illness, may inform society’s ethics in treating those with schizophrenia as well as society’s response to this type of difference (Elder, 1996; Walker, 2019).

Thus, as an ethical lens, LCT provides a method to check one-self—to perform an “ethical check-in” to ensure that ethical principles are scrupulously followed. Just as individuals diagnosed with Alzheimer’s disease may exhibit difficulties in orientation, memory and speech (Alzheimer’s Association, 2021; Perpetuini et al., 2020), the same symptoms are found in older adults aging with schizophrenia, who also have issues with memory and orientation as well as speech organization and coherence (American Psychiatric Association, 2013). In etiology as well disease outcomes, Alzheimer’s disease and schizophrenia differ, but genetic studies and research on brain matter have identified similarities in the two diseases’ processes (Kochunov et al., 2021; Stepanov et al., 2017). Therefore, standardized methods for ethical interaction with older adults with Alzheimer’s disease may also be appropriate in mental health nursing practice and research with older adults aging with schizophrenia (Allison-Kolb, 2020). The Alzheimer’s Society’s principles of person-centered care for those with limited cognition due to dementia or major neurocognitive disorder fit well with older adults aging with schizophrenia. Such a person-centered approach has implications for mental health nursing practice and nursing research with this population. Given a person-centered model, an individual’s history and unique experiences can be seen to inform mental health clinical and research interactions and experiences, and Elder’s model enables one to organize and explain those experiences along the lifespan (Alzheimer’s Society, 2021; Elder, 1996; Elder & Giele, 2009; Fazio et al., 2018; Walker, 2019). With the use of both models, individuals asked to participate in mental health nursing care plans or nursing research become more than just older adults aging with medical conditions. Their past, their unique experiences, and their own cultures and preferences become recognized.

Together with Elder’s LCT, the person-centered approach for those with dementia or major neurocognitive disorder provides an ethical lens for self-examination as nurses engage with older adults aging with the diagnosis of schizophrenia in clinical practice and/or research.

1. Treating individuals with dignity and respect; creating mutually beneficial relationships that involve *doing with*, rather than *doing for*, others (Alzheimer’s Society, 2021; Fazio et al., 2018). In nursing research and mental health nursing practice, this means that older adults aging with schizophrenia will be treated with dignity and respect. Although mannerisms of such individuals may sometimes appear odd or unusual (American Psychiatric Association, 2013), nurses in mental health nursing practice and nursing research should strive to value each patient’s uniqueness and reduce the negative effects of stigma by using words and deeds that promote self-esteem and show acceptance (Morgades-Bamba et al., 2019; Okhakhume, 2012). Nurses’ nonverbal and paraverbal behaviours can be interpreted as greater indicators of how they feel than their verbal communications, and these behaviours therefore require self-monitoring (Wanko Keutchafo et al., 2020; Windle et al., 2020). A person-centered approach that promotes dignity and respect encourages independence in older adults aging with schizophrenia instead of dependence whenever this is possible in the administration of mental health nursing practice and nursing research. Older adults report feeling respected when their independence is encouraged (Koskenniemi et al., 2015). Elder’s concepts of *human agency and social constraints* and *linked lives* (Elder, 1996; Elder & Giele, 2009) align with a person-centered promotion of dignity and respect, with *doing with* rather than *doing for*. Given an awareness of *human agency and social constraints*, the mental health nurse and nurse researcher acknowledge the rights of older adults aging with schizophrenia to express their autonomy regarding their willingness to participate or not participate in mental health nursing clinical procedures or research projects. Those rights include the right to determine whether or not they feel comfortable during the clinical procedure or the research process. Thus, with respect to research, the right of autonomy might include deciding not to participate in a study or stopping to participate before the study is completed, owing to various factors. Nursing researchers and mental health nurses must scrupulously support and respect this autonomy (Doody & Noonan, 2016; White, 2020). With respect to *linked lives*, the mental health nurse and the nursing researcher acknowledge that the lives of key people intersect with the lives of older adults aging with schizophrenia along their life course; this includes the lives of nurses (Elder, 1996; Elder & Giele, 2009; Walker, 2019). Linking lives with older adults aging with schizophrenia in a research study or clinically in a mental health facility may be a very important experience for them, because their interpersonal experiences have been limited along the life course (Burbach, 2018; Fakorede et al., 2020; Robustelli et al., 2017). Some older adults aging with schizophrenia may find it difficult to reach the end of their participation in a research study or a mental health treatment plan, because this experience of linked lives is personally important (Walker, 2020). Older adults aging with schizophrenia must be thoughtfully prepared for the conclusion of a treatment plan or a research study by frequently communicating how long the plan or study will take and when it will end. These facts may need to be mentioned repetitively, owing to potential limitations resulting from older adults aging with schizophrenias’ cognitive states (American Psychiatric Association, 2013; Avery et al., 2020).
2. Using the history, lifestyle, unique experiences, values, preferences and culture of the individual to inform all interactions and experiences; considering the person as more than just a diagnosis (Alzheimer’s Society, 2021; Fazio et al., 2018; Zolnierek, 2007). Every adult aging with schizophrenia is unique, with specific life experiences. How older adults aging with schizophrenia have interacted with their environment over the life course will affect how they perceive their involvement as a participant in a mental health treatment plan or in a nursing research study. A person-centered approach can enable the mental health nurse or nurse researcher to view the older adult aging with schizophrenia as a worthy, whole person, not just as a mental health patient or an older adult with a diagnosis who provides study results (American Holistic Nurses Association, 2021). With a person-centered approach, the nurse researcher and mental health nurse can listen actively and be aware of body language or paraverbal expressions by older adults aging with schizophrenia, which may be strong indicators of how they are feeling (Tennant et al., 2020). The principles of person-centered research enable the nurse researcher and mental health nurse to recognize each individual participant and mental health patient as singular and valued (Alzheimer’s Society, 2021; Fazio et al., 2018). This individuality is in part the result of the person’s unique culture and life journey. Emotional reactions or responses of older adults aging with schizophrenia during research or during a mental health clinical treatment may be related to the individual’s cultural experiences of stigma, shame, isolation, trauma and stress along the life course (Al Jurdi et al., 2014; Burbach, 2018; Cancel et al., 2019; Fakorede et al., 2020; Gauillard, 2016; Ogden, 2014a, 2014b; Robustelli et al., 2017; Ruby et al., 2017; Walker, 2019; Zimmerman et al., 2016). Consideration, thoughtfulness and patience related to these factors take precedence over the details of obtaining data, or time lines associated with the accumulation of those data. Elder’s concept of *human lives in historical time and place* thus also aligns with a person-centered approach (Elder, 1996; Elder & Giele, 2009). Individuals born at the same time share historical or societal events that they have experienced; they live within similar circumstances that yield *cohort effects*. For example, individuals currently aging with schizophrenia are likely to have experienced deinstitutionalization or transinstitutionalization (Geller, 2000; Kim, 2016). The current perceptions that older adults aging with schizophrenia have of society, which can include their perceptions of researchers and mental health nurses, may be influenced by how they have learned to trust or mistrust those who have been involved in their societal care (Walker, 2019). When they were younger, these older adults may have experienced a medical model of care. For them, research or clinical care that is person-centered, in which they are considered the centre of the care or as partners in the care or research, may present a new model (Graffigna & Barello, 2018). The idea that older adults aging with schizophrenia have a say, or that they can participate with distinct rights, may not be what they have been accustomed to. This is an important factor for research and mental health clinical treatments with this population. The right to full consent and the knowledge that the older adult can say “no” and terminate involvement in mental health clinical treatments and research at any time must be presented clearly. This is especially essential in communicating with those who are vulnerable due to limited cognitive abilities in mental disorders such as schizophrenia (American Psychiatric Association, 2013; Doody & Noonan, 2016; White, 2020).
3. Looking at situations with empathy or from the individual’s point of view; accepting others’ reality and trying to listen to their speech and/or behaviours in an accepting, validating manner (Alzheimer’s Society, 2021; Fazio et al., 2018; Kerasidou, 2020). The mental health nurse and the nurse researcher should take time to listen to the stories of older adult aging with schizophrenia and may, with their permission, report those stories to society, which can be a validating experience for these older adults (Bartolucci & Batini, 2019; Lee, 2016; Ogden, 2014a, 2014b). The stories of older adults aging with schizophrenia are often unheard, in part because of their sometimes limited ability to communicate with others, which can result from schizophrenia’s negative or positive symptoms (American Psychiatric Association, 2013; Riehle et al., 2018). Communication with older adults aging with schizophrenia may not always be clear, and they may use strange sounds and nonverbal gestures in communicating their stories because of limited cognitive abilities. Or they may isolate themselves and engage in little to no communication, owing to manifestations of negative symptoms (American Psychiatric Association, 2013; Liu et al., 2020). Taking the time to listen with empathy regardless of what is said or not said by the older adult is a way to apply a person-centered approach within the context of mental health nursing practice and nursing research. What older adults aging with schizophrenia offer to the mental health nurse or the nursing researcher must be valued and respected, even when it may seem limited (Lee, 2016; Liu et al., 2020). Elder’s *linked lives* thus also aligns with a person-centered approach. Older adults aging with schizophrenia have intersected with many different individuals along their life course. Some of these linked lives may have provided support and kindness, but many may not have done so and may instead have promoted the stigma that often follows the older adult aging with schizophrenia. This stigma and its effect along the life course can affect how the older adult with schizophrenia views mental health clinical treatments and the research process (Morgades-Bamba et al., 2019; Walker, 2019). It can be stigmatizing to be the subject of research or to engage with others in group therapies—to share intimate details of hearing voices or having delusions, none of which are common experiences. Such unique experiences can lead to name calling; older adults aging with schizophrenia are often labelled “odd” or “crazy” (Goodwin & Tajjudin, 2016; Schulze et al., 2003). Their perceptions of being stigmatized may make it difficult for them to be fully honest in sharing their life experiences. In addition, the diagnosis of a stigmatized illness may be associated with depression and behavioral isolation (Brooks et al., 2019; Penkunas et al., 2015). In research and clinical practice, both confidentiality and privacy can prevent further stigmatization that might lead to increased depression, affecting both morbidity and mortality (Touriño et al., 2018; Yanos et al., 2008).
4. Helping the individual to try new things, have choices, be involved and engaged in shared activities that are enjoyable, with chances for autonomy; recognizing that individuals may continue to experience joy, meaning, and comfort in life as they age (Alzheimer’s Society, 2021; Fazio et al., 2018; Steenbergen, 2021). A person-centered approach can encourage the choices of older adults aging with schizophrenia to engage in research opportunities and mental health clinical activities if participation is something that they view as joyful and meaningful. The sharing of their voices and life experiences might be a meaningful exercise that enables them to communicate with society about their healthcare experiences. For some, it might simply be an enjoyable experience of sharing and engaging with the mental health nurse or the nurse researcher. Elder’s *human agency and social constraints* and *the timing of lives* (Elder, 1996; Elder & Giele, 2009) align with this aspect of a person-centered approach to research. *Human agency and social constraints* address how individuals aging with schizophrenia are given the freedom to make choices regarding the course of their lives. Past experiences of social freedom or lack thereof might influence how older adults aging with schizophrenia view their autonomy when they consider participating in mental health clinical treatments and/or research. The past history of vulnerability and lack of freedom among research participants can suggest not only that older adults aging with schizophrenia need to be protected but also that their involvement in research studies might not be to their advantage (Doody & Noonan, 2016; White, 2020). *Safety* may seem more important than autonomy. However, older adults aging with schizophrenia may wish to exercise autonomy when considering involvement in research projects. If so, their desire to participate in research must be respected and supported (Steenbergen, 2021). The principle of *the timing of lives* acknowledges that older adults aging with schizophrenia may not have had the opportunities to marry, have children, go to school or be employed as have many others in their same age group. Involvement with nursing researchers and mental health clinical nurses may be an opportunity for older adults aging with schizophrenia to engage in meaningful relationships that they have not always had the opportunity to experience along their life course (Walker, 2019).

## 3 | RECOMMENDATIONS

Nurses conducting research and/or engaging in mental health practice with older adults diagnosed with schizophrenia must be aware that the cognition of these patients may preclude them from immediately understanding informed consent for research or medical procedures (American Psychiatric Association, 2013). Other limiting factors to ethical nursing practice and research with older adults diagnosed with schizophrenia include physiological issues such as potentially poor eyesight or hearing problems (Tian et al., 2021; Walker, 2021). Thus, a meticulous method for obtaining informed consent might be necessary when engaging this population in nursing research and practice. The use of iterative feedback in which the individual repeats back in their own words the meaning of the procedure or study is recommended for this population as an ethical practice. Open-ended questions should be asked of the individual about the informed consent procedure. The individual’s ability to understand will be defined by their ability to accurately respond to these questions posed by the nurse researcher or mental health nurse (Stiles et al., 2001).

Other recommendations include extending the umbrella of veracity to include not only the older adult diagnosed with schizophrenia but also the researcher or/and mental health nurse. “Ethical check-in” questions could include the following:

1. Do I honestly believe my intersection with this person is beneficial and non-harmful?
2. Do I honestly believe that the older adult diagnosed with schizophrenia understands the procedure or research intervention and has autonomously chosen to participate?
3. Am I willing to be vigilant with respect to the vulnerability of this person and change the course of my intervention if I become aware that a positive interaction has turned negative?

The ability of a vulnerable person such as an older adult diagnosed with schizophrenia to participate in research and mental health practice interventions and exercise their autonomy is linked to the veracity and non-maleficence of the health care providers who are entrusted with their care.

## 4 | DISCUSSION

The lens of LCT with a person-centered approach offers a method with which nursing researchers and mental health clinical nurses can self-check their practices for ethics in their research and clinical practice with vulnerable populations such as older adults aging with schizophrenia. Table 1 illustrates the ethical principles of veracity, non-maleficence, vulnerability and autonomy according to the model, with the desired end of conducting ethical nursing research and ethical mental health nursing practice. Other ethical principles, however, could be entered into the model with equal success. The model is constructed to generate questions for nurse researchers and mental health nurses in clinical practice about older adults aging with schizophrenia and their life experiences, in order to thoughtfully predict how these older adults might potentially respond to the research process and mental health treatment plans. It is also set up to generate questions about the nurse’s practices, enabling the nurse to check in to see whether the nurse’s actions are patient centered, kind and thoughtful, with the participant/patient viewed as a worthy human being, not just a research subject or mental health patient. LCT, combined with a person-centered approach, has the potential to increase the likelihood that nursing research and mental health nursing practice with vulnerable populations such as older adults diagnosed with schizophrenia will be conducted ethically.

## 5 | RELEVANCE FOR CLINICAL PRACTICE

The use of LCT with a person-centered approach can encourage nurses in research and mental health nursing practice to seek important information collaboratively with older adults diagnosed with schizophrenia in a thoughtful and ethical manner, to inform improvement of their health outcomes as well as health policy. Nursing research and mental health nursing practice with an LCT/person-centered lens can help nurses to thoughtfully envision, explore and generate interventions to address the special needs of older adults aging with schizophrenia, working together with them toward patient-centered goals and objectives.

## Figures and Tables

**TABLE 1:** Delineates a self-check of practices for ethics in nursing research and clinical practice with vulnerable populations such as older adults aging with schizophrenia

Ethical Principle to be examined by nursing researchers and clinical nurses	Elder’s life course theory lens	Patient-centered care lens	Questions generated after examination of both lenses	Desired result
Veracity	Linked Lives: Are nurse researchers and mental health clinical nurses (linked lives) intersecting truthfully with older adults aging with schizophrenia truthful?	Those who practice person-cenrred care will listen actively and be aware of body language or paraverbal expressions by older adults aging with schizophrenia, which may be strong indicators of how they are feeling	Has the research study or/and nursing intervention been explained in a clear, honest, understandable manner to older adults aging with schizophrenia?	Ethical nursing research and ethical mental health clinical nursing practice with older adults aging with schizophrenia
Non-maleficence	Human Lives in Historical Time and Place: How have past societal experiences affected older adults aging with schizophrenia in how they interact with others now?	Those who practice patient-centered care will use history and preferences to inform all interactions with older adults aging with schizophrenia.	Have older adults diagnosed with schizophrenia had negative interactions/experiences with health care providers in the past that might influence the way they view their interviews with nurse researchers/nurse clinicians?	Ethical nursing research and ethical mental health clinical nursing practice with older adults aging with schizophrenia
Vulnerability	The Timing of Lives: Timing for older adults aging with schizophrenia may be different than it is for others in society regarding education, employment, and marriage. Do current intersections promote trust and safety for older adults aging with schizophrenia?	Those who practice person-centered care will view situations with empathy or from the point of view of older adults aging with schizophrenia, accepting their reality and trying to listen to their speech and/or behaviours in an accepting, validating manner	Is the vulnerability of older adults aging with schizophrenia being considered to promote their safety in both nursing research and mental health nursing practice?	Ethical nursing research and ethical mental health clinical nursing practice with older adults aging with schizophrenia
Autonomy	Human Agency and Social Constraints: Are individuals aging with schizophrenia given the freedom to make choices regarding the course of their lives?	Those who practice person-centered care will encourage older adults aging with schizophrenia to try new things, have choices, be involved and engaged in shared activities that are enjoyable, with chances for autonomy	Is the autonomy of older adults aging with schizophrenia being respected regarding their desire to participate in nursing research and mental health nursing practice?	Ethical nursing research and ethical mental health clinical nursing practice with older adults aging with schizophrenia

## Data Availability

Data sharing not applicable – no new data generated, or the article describes entirely theoretical research.
